# Evaluating the Perceived Benefits and Effectiveness of a Government Health Insurance Scheme for Chronic Kidney Disease Patients: A Study in Nellore, Andhra Pradesh

**DOI:** 10.7759/cureus.75631

**Published:** 2024-12-13

**Authors:** Shanmukh P Annadata, Sneha Ragupathy, Rekha S, Tanya S Isaac, Gayathri KG, Vidhya Venugopal

**Affiliations:** 1 General Medicine, Sri Ramachandra Medical College, Chennai, IND; 2 General Medicine, American University of Antigua, Osbourn, ATG; 3 Department of Environmental Health Engineering, Faculty of Public Health, Sri Ramachandra Institute of Higher Education and Research (Deemed to be University), Chennai, IND

**Keywords:** access to healthcare, challenges, ckd, effectiveness, health insurance scheme, perceived benefits

## Abstract

Background

Chronic kidney disease (CKD) is prevalent in India, particularly among underprivileged populations. Government initiatives such as the Dr. YSR Aarogyasri Health Insurance Scheme aim to provide affordable healthcare to economically impoverished individuals with kidney diseases. This study assessed participants’ perceptions of the scheme’s benefits, effectiveness, and challenges.

Methods

A cross-sectional study was conducted in Nellore, Andhra Pradesh, involving 100 CKD patients. Data was collected using a structured questionnaire assessing socio-demographic factors, insurance scheme’s awareness, perceived benefits, and effectiveness. Multivariable logistic regression analysis was employed to identify factors influencing participants’ perceptions.

Results

Participants aged over 40 years were more likely to perceive the Dr. YSR Aarogyasri Health Insurance Scheme positively (crude odds ratio {COR}: 9.2; 95% CI: 2.6-31.7). Males (adjusted odds ration {aOR} 1.5; 95% CI: 1.1-2.1), married individuals (aOR 1.3; 95% CI: 0.8-1.7), and those with higher education (aOR 1.9; 95% CI: 1.1-3.2) reported greater scheme effectiveness. However, 32% of CKD patients lacked awareness of the scheme’s enrolment process, 29% believed that the eligibility criteria were too strict, and 17% of insured patients reported gaps in coverage for specific services, treatments, and medications, reducing the scheme’s appeal to diseased patients.

Discussion

Older males and those with diabetes, who require extensive care, perceive the scheme as beneficial. However, gaps in awareness and coverage remain barriers to its full effectiveness.

Conclusion

While the Dr. YSR Aarogyasri Health Insurance Scheme provides critical financial relief for CKD patients, enhancing its outreach and expanding coverage could improve its impact. These findings can guide policy improvements to optimize the scheme’s effectiveness and accessibility.

## Introduction

Chronic kidney disease (CKD) is the irreversible, progressive deterioration of renal function over time [1]. CKD affects 17.2% of the Indian population, indicating a significant public health problem. Projections indicate that the disease burden will continue to rise, emphasizing the need for effective prevention and management strategies [2]. While hemodialysis (HD) is an effective treatment, its high cost and complexity restrict access to high-quality healthcare for the majority of patients in India [3]. Consequently, individuals from lower socioeconomic backgrounds are disproportionately affected, leading to significant health and social repercussions [4]. Annually, approximately 1.4 million people in India lose their lives, primarily due to the inaccessibility of HD resources [5].

The financial repercussions for a household can be significant when an individual becomes ill and is burdened with the upfront costs of medical treatment without robust social safety nets, families may face financial hardship due to medical expenses, lost wages due to missed work, disability, or even premature death, which can lead to a decline in their overall income. Insurance can help mitigate this cost for a family by reducing their out-of-pocket health expenses and decreasing the likelihood of financial hardship and risk of impoverishment due to illness [6]. While health insurance coverage may increase healthcare utilization and out-of-pocket expenses, it generally provides greater access to care and improves both health and economic outcomes for households [7]. This underscores the importance of providing sufficient healthcare services and financial protection, particularly for individuals requiring HD due to CKD. Health insurance schemes play a critical role in guaranteeing that these patients have access to affordable and comprehensive healthcare [8]. A variety of healthcare programs offered by the central and state governments, non-governmental organizations (NGOs), and hospitals can help address these needs.

In India, various healthcare schemes have been developed to provide financial protection and improve access to quality care, particularly for vulnerable populations. Two largely successful programs include the Pradhan Mantri Jan Arogya Yojana (PMJAY), which provides health insurance coverage of up to five lakh rupees annually for economically disadvantaged families, and the Rashtriya Swasthya Bima Yojana (RSBY) that offers insurance to families living below the poverty line (BPL), particularly in rural areas [9]. One such initiative in the state of Andhra Pradesh is the Dr. YSR Aarogyasri Health Insurance Scheme, which was launched by the then Chief Minister of Andhra Pradesh, Dr. YS Rajasekhara Reddy in 2007 [10]. The scheme has two primary objectives: to provide financial security in the event of escalating medical expenditure, and the second is to ensure quality healthcare for the economically disadvantaged [11]. Since its inception, the scheme has extended coverage to 1059 surgical interventions and 30 medical procedures [11]. Eligibility is primarily based on having a BPL card, an Annapurna card, or ration cards from the Anthyodaya Anna Yojana program, as well as for individuals suffering from certain medical conditions. With 86% of Andhra Pradesh’s population holding BPL cards, the scheme has a broad reach. It offers up to five lakh rupees per household per year in healthcare expenditure, providing crucial support to vulnerable populations [7].

The Dr. YSR Aarogyasri Health Insurance Scheme is unique in its implementation, considering Andhra Pradesh is the only state government that provides universal health care to the poor [12]. It allows patients to receive treatment at their preferred hospitals and uses web-based processing to ensure transparency across all stages, including health camp participation, screening, diagnosis, treatment, follow-up, and claim settlement, effectively preventing misuse and fraud [11]. By combining the facilities provided by government hospitals, Dr. YSR Aarogyasri Health Insurance Scheme grants the BPL population universal health coverage, including preventative care, primary care, and inpatient treatment. Insurance schemes such as this will provide financial protection to vulnerable populations in the event of a health shock, helping to reduce catastrophic out-of-pocket expenses, and ensuring healthcare as a fundamental human right [12].

The Screening and Early Evaluation of Kidney Diseases (SEEK) study identified districts in Andhra Pradesh as having some of the highest rates of CKD in the country [2]. Given this significant disease burden, it is imperative that health insurance schemes effectively detect and treat CKD [3]. Understanding enrolment, utilization, and adherence to optimal practices by HD patients under such schemes is essential to improving healthcare delivery and identifying opportunities for growth. Further research is required to evaluate the program’s impact and explore the potential benefits of implementing similar schemes nationwide, particularly for the BPL population. Collecting data and identifying gaps in the program will be instrumental in enhancing the program’s effectiveness. This study aimed to assess participants’ perceptions of the scheme's impact on financial security and access to high-quality HD treatment, while also examining the benefits, effectiveness, and challenges of utilizing this healthcare provision.

## Materials and methods

Study design and setting

We used a structured questionnaire to do a cross-sectional pilot study in the district of Nellore, Andhra Pradesh, India, from March to November 2023 to find out how people with CKD felt about the Dr. YSR Aarogyasri Health Insurance Scheme and how well they thought it worked. The district is one of the nine coastal districts of Andhra Pradesh, with a population of 32.9 lakh individuals, the majority of whom speak Telugu as their native language. This area has a high prevalence of CKD and has several beneficiaries for the Dr. YSR Aarogyasri Health Insurance Scheme [13].

Data collection

We conducted this study on 100 individuals diagnosed with CKD who sought treatment, including dialysis, at a tertiary care hospital in Nellore. We collected data on volunteered participants who visited the hospital for treatment during a specific period, which is the rationale for the smaller sample size. The sample size was calculated using the formula n = [Z^2^ * P * (q)]/d^2^. The derived sample size was 96, and it was rounded off to 100. The study included male and female participants aged between 21 and 80 years. We selected participants based on their willingness to participate, and each individual provided written informed consent prior to participation. The Institutional Ethics Committee, Sri Ramachandra Institute of Higher Education and Research, Chennai, granted ethical clearance (Approval No.: IEC-NI/17/APR/59/54).

We used a validated and structured questionnaire for data collection, which experts translated into Telugu. We pretested the questionnaire for reliability and validity. The Cronbach value is α=6.4. After making sure the tool was valid and reliable, a group of nephrology and public health experts approved it. We also collected information from patients’ case sheets and family members. We developed the questionnaire by reviewing existing literature and consulting experts to cover all relevant aspects of CKD and the Dr. YSR Aarogyasri Health Insurance Scheme. Competent experts incorporated feedback from the experts into the final questionnaire and translated it into Telugu. We undertook necessary revisions to enhance the instrument's clarity and relevance. The questionnaire evaluated awareness, knowledge, benefits, perception, utilization, best practices, and challenges faced by people with CKD enrolled in the Dr. YSR Aarogyasri Health Insurance Scheme (see Appendices).

We divided the questionnaire into five parts. (1) Part A: Demographic variables such as age, sex, educational status, occupation, marital status, BMI, presence of comorbidities (diabetes and hypertension), and history of alcohol intake and smoking. (2) Part B: Detailed HD information, including duration, frequency, and distance traveled for treatment. (3) Part C: Perception of CKD health schemes. (4) Part D: Preferences regarding CKD health schemes. (5) Part E: Evaluation of the scheme’s effectiveness and the challenges in accessing the benefits.

Key questions explored the perceived benefits provided by the scheme, the effectiveness of treatment of the scheme, and the challenges encountered in accessing the scheme. The following are a sample of questions included. (1) Do you have knowledge about the CKD health schemes rolled out by the government? (2) Do you believe that utilizing CKD health schemes could prevent financial hardship in managing your CKD? (3) Is the CKD health scheme beneficial and simple to access? (4) CKD health scheme benefits are higher than the cost of HD treatment (rating). (5) Is this scheme's enrolment process convenient for you? (6) How would you rate your insurance benefits? (rating) (7) How easy do you feel about managing your overall health after using the scheme? (rating) (8) In your opinion, which is more beneficial and effective: the government health insurance scheme or private health insurance? (9) What options do you think could improve the use of the scheme in the state? (10) What are the challenges you face in accessing the scheme?

When the response was "no," we assigned a score of 0 for perceived benefits, and a score of 1 for "yes." Rating scale questions used a 5-point Likert scale ranging from “strongly disagree" to “strongly agree,” with responses coded from 1 to 5 based on the participants’ responses for both perceived benefits and effectiveness sections.

Data analysis

The study mostly looked at two outcomes: how people with CKD felt about the Dr. YSR Aarogyasri Health Insurance Scheme's benefits and how well it worked for them. Perceived benefits refer to the participants’ perception of financial relief and improved access to healthcare services through the scheme, while effectiveness focuses on the extent to which the scheme met their expectations for treatment and healthcare access.

To analyze these outcomes, we first created two new variables: perceived benefits and effectiveness, based on participants’ total scores. We calculated the total scores of participants for both perceived benefits and effectiveness and determined the median score. Participants were categorized based on whether their total scores were above or below the median. For perceived benefits, scores equal to or above the median were considered "high," and below the median were considered "low." The median score also determined the categorization of effectiveness as "effective" or "ineffective". The analysis treated these stratified categories as dependent variables.

Next, we conducted an analysis of participants’ knowledge of the Dr. YSR Aarogyasri Health Insurance Scheme. This model assessed how well participants understood the scheme and whether they believed it offered benefits. To take into account possible confounders, we changed a number of health and sociodemographic factors, such as age, gender, marital status, level of education, occupation, alcohol and smoking history, and having a comorbidity (diabetes and high blood pressure). We used logistic regression to investigate the relationship between the two stratified outcome variables and the participant's knowledge of the scheme.

We then performed a demographic analysis, considering how other demographic variables influenced the participants’ perceived benefits and effectiveness of the scheme. We calculated the adjusted odds ratios (aOR) using a multivariate logistic regression (MLR) model. All data were entered into Microsoft Excel (Microsoft Corp., Redmond, WA) and statistical analysis was conducted using R Studio software, version 4.3.1 for Mac (The R Foundation for Statistical Computing, Vienna, Austria).

## Results

Socio-demographic characteristics of study participants

Of the 100 participants surveyed, approximately three-fourths of the participants (76%) were more than 40 years of age, and 81% of them were male. Among the participants, 88% were married. Less than half of them (40%) had attained an education from a higher-secondary school or above. Regarding substance consumption, approximately a fifth (19%) indicated a history of smoking, while half (47%) indicated a history of alcohol consumption. Agriculture and service/business employed 65% of the participants, while 35% were unemployed. The full breakdown of demographic characteristics can be found in Table 1.

**Table 1:** Socio-demographic characteristics of participants from Andhra Pradesh, Southern India (n = 100).

S. No	Characteristics	Percentage (n = 100)
1	Age (years)	
	≥40	76
	<40	24
2	Sex	
	Male	81
	Female	19
3	Marital status	
	Married	88
	Unmarried	12
4	Education level	
	Higher-secondary and above	40
	Primary/secondary and below	60
5	Smoking status	
	Yes	19
	No	81
6	Alcohol consumption	
	Yes	47
	No	53
7	Occupational status	
	Service/business	32
	Agriculture	33
	Unemployed	35

Knowledge, perceived benefits, and effectiveness of the Dr. YSR Aarogyasri Health Insurance Scheme

A total of 86% of participants were aware of the Dr. YSR Aarogyasri Health Insurance Scheme, while 14% were not informed. As can be seen in Table 2, the logistic regression analysis showed that people who knew more about the scheme were 1.5 times more likely to think it was helpful (COR: 1.5, 95% CI {0.4-5.8}). Additionally, those who perceived the scheme as effective were predominantly those already informed about it (90.7% vs 100% in the uninformed group).

**Table 2:** Bivariate logistic regression model assessing the association between knowledge of the Dr. YSR Health Insurance Scheme and its perceived benefits and effectiveness among chronic kidney disease participants (n = 100). An odds ratio higher than 1 denotes the presence of risk; p-value <0.05 shows significance; this analysis was adjusted for age, sex, education level, presence of comorbidities, alcohol consumption, and smoking status. COR: crude odds ratio.

Parameters	Percentage of participants informed of the Dr. YSR Health Insurance Scheme (n = 86)	Percentage of participants uninformed of the Dr. YSR Health Insurance Scheme (n = 14)	COR (95% CI)
Perceived benefits			
Low	29.1 (25)	21.4 (3)	1.5 (0.4-5.8)
High	70.9 (61)	78.6 (11)	1.00
Effectiveness			
Ineffective	9.3 (8)	0	0.9 (0.1-0.9)
Effective	90.7 (78)	100 (14)	1.00

From the MLR analysis, as outlined in Table 3, certain socio-demographic factors were associated with better perceptions of the scheme’s benefits and effectiveness. Participants who were male (aOR 1.7; 95% CI {0.9-2.7}), married (aOR 1.3; 95% CI {0.8-1.7}), and had a higher secondary education or more (aOR 1.3; 95% CI {0.8-1.9}) were more likely to perceive the program as helpful and effective (aOR 1.5; 95% CI {1.1-2.1}), (aOR 1.2; 95% CI {0.9-1.5}), and (aOR 1.9; 95% CI {1.1-3.2}) compared to the reference group (female), unmarried, and those with only a primary or secondary education or less. Participants who were employed in a service or business were less likely to perceive benefits (aOR 0.9; 95% CI {0.6-1.4}] or find the scheme effective (aOR 0.9; 95% CI {0.7-1.2}) when compared to participants engaged in agriculture or working in informal sectors. Participants who had a history of drinking and smoking were less likely to think that the program was more helpful (aOR 0.7; 95% CI {0.5-1.1}), more effective (aOR 0.9; 95% CI {0.7-1.2}), or less likely to think that it was effective (aOR 0.8; 95% CI {0.6-1.2}) than people who had never drunk or smoked.

**Table 3:** Multivariable logistic regression model assessing the association of perceived benefits and effectiveness of the YSR Health Insurance Scheme in chronic kidney disease participants by socio-demographic characteristics (n = 100). An odds ratio higher than 1 denotes the presence of risk. COR: crude odds ratio, aOR: adjusted odds ratio.

Parameters	Perceived benefits		Effectiveness of undergoing the scheme	
	COR (95% CI)	aOR (95% CI)	COR (95% CI)	aOR (95% CI)
Age (years)				
<40	1.6 (0.6-4.2)	0.8 (0.6-1.7)	0.4 (0.1-4.3)	0.9 (0.7-1.2)
>40	1.00	1.00	1.00	1.00
Sex				
Male	2.1 (0.6-6.2)	1.7 (0.9-2.7)	14.8 (7.1-31.3)	1.5 (1.1-2.1)
Female	1.00	1.00	1.00	1.00
Marital status				
Married	1.8 (0.5-5.9)	1.3 (0.8-1.7)	4.1 (0.4-41.1)	1.2 (0.9-1.5)
Unmarried	1.00	1.00	1.00	1.00
Education level				
Higher-secondary and above	0.6 (0.2-1.6)	1.3 (0.8-1.9)	2.8 (0.2-22.7)	1.9 (1.1-3.3)
Primary-secondary and below	1.00	1.00	1.00	1.00
Occupation				
Service/business	0.9 (0.3-2.5)	0.9 (0.7-1.4)	0.8 (0.1-3.4)	0.9 (0.7-1.2)
Unemployed	1.1 (0.3-3.1)	0.9 (0.7-1.4)	0.8 (0.6-3.7)	0.9 (0.7-1.2)
Agriculture	1.00	1.00	1.00	1.00
Alcohol consumption				
Yes	0.5 (0.2-1.5)	0.7 (0.5-1.1)	0.09 (0.1-0.8)	0.9 (0.7-1.2)
No	1.00	1.00	1.00	1.00
Smoking history				
Yes	0.4 (0.1-1.5)	0.9 (0.5-1.4)	1.1 (0.1-3.4)	0.9 (0.6-1.2)
No	1.00	1.00	1.00	1.00
Presence of comorbidities				
Diabetes	9.4 (3.5-25.1)	2.4 (1.5-3.8)	1.2 (1.1-1.9)	1.3 (1.0-1.6)
Hypertension	4.3. (2.3-8.0)	0.9 (0.5-1.5)	0.9 (0.7-1.3)	0.9 (0.7-1.3)

People who had diabetes were much more likely to think the plan was helpful (aOR 2.4; 95% CI {1.5-3.8}) and useful (aOR 1.3; 95% CI {1.0-1.6}). In contrast, those with hypertension were less likely to think that the program was helpful (aOR 0.9; 95% CI {0.5-1.4}) or effective (aOR 0.9; 95% CI {0.7-1.3}) than participants who did not have hypertension.

Challenges in accessing the insurance scheme

While the Dr. YSR Aarogyasri Health Insurance Scheme offers significant financial relief and support, participants with CKD reported encountering few challenges in accessing its benefits. The following are some of the challenges. (1) A significant number of individuals are unaware of the existence of this free government insurance scheme or the process of enrolling in it, as indicated by 32% of the patients. There is a lack of effective outreach and education efforts to further disseminate the presence and utility of such schemes to the general public through media or other sources. (2) The strict eligibility criteria excluded certain individuals or families from participating in the initiative, according to 29% of the patients. For instance, the inability of certain individuals to meet the BPL income thresholds renders the schemes controversial and inaccessible. (3) According to 17% of the patients, the insurance scheme offers limited benefits and excludes specific services, treatments, or medications, leading to coverage gaps that negatively impact insured patients. This further intensifies the already heavy financial burden on the diseased individual and their families.

## Discussion

This study investigated the perceived benefits, effectiveness, and challenges of this scheme for people with CKD undergoing HD treatment. Historically, insurance has not been a viable option for low-income individuals. People assumed that they were too economically disadvantaged to save or pay a premium. Therefore, the government assumed the responsibility of providing healthcare services to households classified as BPL in Andhra Pradesh.

The insurance company under this scheme pays the hospital bills of the insured persons. The government pays the insurance company's premium, leaving people with no financial burden. Health insurance schemes, such as this one, serve as an alternative method of financing healthcare expenses [11]. The main goal of the Dr. YSR Aarogyasri Health Insurance Scheme, which is part of health reform plans and initiatives, is to make sure that the poor and vulnerable people of Andhra Pradesh can get effective and affordable health care [12]. This study looked at the medical and socio-demographic details of the people who took part and found the main things that affected how they felt about the Dr. YSR Aarogyasri Health Insurance Scheme for HD patients. Gender, age, and the presence of additional health conditions, especially diabetes, emerged as significant. Enrolled participants over 40 years were more likely to perceive the scheme as beneficial, possibly due to their increased exposure to television advertising and "information, education, and communication" (IEC) materials during their visits to government hospitals for treatment.

HD patients who already have diabetes found the insurance scheme 1.2 times (p = 0.046) more effective than HD patients without diabetes; male HD patients were 1.6 times (p = 0.042) more likely to find the insurance scheme to be beneficial and 1.5 times (p = 0.013) more effective than their female counterparts. HD patients with diabetes often require comprehensive healthcare services, including medications, regular checkups, and specialized treatments [14]. The Dr. YSR Aarogyasri health insurance scheme provides extensive coverage for diabetes management, including medications, diagnostic tests, and consultations with endocrinologists. This also includes preventative services. HD patients with diabetes may recognize the importance of preventive care in managing their overall health, and since the scheme supports such services, it can contribute to the increased perceived effectiveness of undergoing CKD treatment under the scheme [15].

Patients with diabetes may find the scheme more effective in addressing their specific healthcare needs because of the extensive coverage for the management of their comorbidities. Additionally, the costs associated with managing diabetes and its complications, such as CKD requiring HD, can be substantial [3,16]. A study in rural Karnataka revealed an increase in the prevalence of CKD, hypertension, and diabetes in rural areas [17]. Since 79% of people live in rural areas, more and more people are being diagnosed with multiple illnesses at the same time, like diabetes, and realizing how important health insurance plans are for making care more affordable [18].

When considering that male participants were more likely to find the insurance scheme to be beneficial and more effective than their female counterparts, it is important to note that 81% of the sample was male. Therefore, it is possible that the male population in this sample simply had a higher probability of holding this view. If the proportion of male participants accurately reflected the approximate number of male individuals in Nellore, the data could have yielded more insights. Regardless, it is crucial to emphasize that this finding could still hold significance due to societal norms and gender roles in India, as well as gender disparities in information access [15]. Families consider men as the primary breadwinners and prioritize their health and well-being [19]. Therefore, male patients may perceive the scheme as more crucial for ensuring financial security for their families. However, this is just speculation and requires further study for validation. Also, differences between men and women in access to information and education make it harder for both sexes to understand the scheme's benefits (in Nellore, the male literacy rate was 88.6% and the female literacy rate was 79.5%), and male participants may think it works better [20,21].

This study serves as a valuable foundation for understanding the preferences and perceptions of Andhra Pradesh's CKD community enrolled in the Dr. YSR Aarogyasri Health Insurance Scheme. These findings highlight the significance of considering diverse sociodemographic characteristics when analyzing health data and formulating targeted interventions for this population. While HD is an effective treatment option for CKD and end-stage renal disease (ESRD), its expense is a substantial barrier for those undergoing it [22]. The challenges faced by the participants in accessing the scheme’s benefits form a basis for policy changes in the implementation of such schemes, not only in India but in other low- and middle-income countries (LMICs) [23]. The efforts to address these challenges include simplifying the application process, enhancing outreach and education, improving coordination services, providing support for non-digital applications, and ensuring that coverage is comprehensive and accessible [24].

This study has a few limitations. First, the conduct of this study in a single private tertiary care hospital in Nellore, along with its small sample size of 100, raises concerns about the broader applicability of its findings. Even the study's confidence interval (CI) is significantly wide, indicating imprecise estimates and suggesting a need for a larger epidemiological study with an increased sample size. Longitudinal data could provide a more comprehensive understanding of the dynamics between HD patients and the insurance scheme over time [25]. Second, we understand the importance of achieving gender balance in the study; unfortunately, increasing the sample size is not feasible at this time. Due to constraints such as limited resources, time, or the availability of eligible participants, we are unable to expand the sample further. However, we will continue to analyze the data as thoroughly as possible and take into account the current sample's limitations when drawing conclusions. The lack of research on this topic highlights a need for more research to fully understand the lower socioeconomic status of people with CKD’s experiences in a universal healthcare insurance program. Even though the study has certain limitations, it is still a useful starting point for learning about how healthcare works in Andhra Pradesh with this representative sample. It also highlights the need for further research to address these issues and investigate the impact of health insurance on a broader spectrum of individuals.

## Conclusions

CKD is on the rise, not only in India but also globally. The Dr. YSR Aarogyasri Health Insurance Scheme, a critical initiative in Andhra Pradesh, India, aims to address the inaccessibility of healthcare for individuals of low socio-economic status. It has parallels to similar initiatives in other LMICs, aimed at safeguarding community health. The findings of this study provide valuable insights into the scheme's impact on CKD patients and lay the foundation for future research and policy reforms in Andhra Pradesh. Building on the early successes of this scheme, the government has an opportunity to enhance the quality of care, foster equitable access to healthcare services, expand the list of covered services, increase the funding for treatment procedures, and target unserved populations through community health programs. Given India's socio-demographic heterogeneity, improving healthcare accessibility and resource distribution remains a critical priority. We must view universal healthcare as a necessity, especially as chronic diseases continue to rise in both urban and rural areas. The results of this study can help policymakers improve the implementation strategies of Dr. YSR Aarogyasri Health Insurance Scheme so that they better meet the healthcare needs and views of different groups of people. Moreover, other LMICs implementing similar government health initiatives can benefit from the insights drawn from this research to improve their own healthcare systems and ensure broader, more inclusive access to vital services.

## Appendices

Questionnaire on awareness of government-initiated CKD health schemes: perception from CKD patients

Part A: Demographic Variables

Name: 

Age: 

Sex: 

Contact no.

Address: 

Profession: 1) farming 2) business 3) government employee 4) others, mention:

Education: 1) illiterate 2) primary school 3) high school 4) graduate

Marital status: 1) married 2) unmarried 3) divorce 4) widow

Annual household income: 1) < 1 lakh 2) 1-2 lakhs 3) 2-5 lakhs 4) > 5 lakhs 

Diagnosis: 

DM: 1 Yes / 2 No 

HTN: 1 Yes / 2 No 

Renal biopsy done: 1 Yes / 2 No if yes, diagnosis:

Family history of kidney disease: 1 Yes / 2 No 

Weight (in kg): 

Height (in cm): 

BMI:

h/o smoking: 1 Yes / 2 No 

h/o alcohol intake: 1 Yes / 2 No 

h/o painkiller use: 1 Yes / 2 No

Number of members in family:

Part B: Hemodialysis Details

HD initiation date:

Duration of dialysis: 1) < 1 yr 2) 1-3 yr 3) 3-5 yr 4) > 5 yr 

Frequency of HD: duration of HD: 

Distance traveled for dialysis: 1) < 5 km 2) 5-10 km 3) 10-20 km 4) > 20 km

Receiving govt. pensions: 1 Yes / 2 No 

If yes, pension details 

Part C: Perception of CKD Health Schemes

1. Do you have knowledge of the CKD health scheme? 1 Yes / 2 No 

a. If yes, how did you come to know

a. Friends/colleagues/supervisors/any others 

b. Magazines, media (TV/radio, etc.)

c. From forefathers 

d. Researchers like us 

e. Others

2. Do you believe that utilizing the CKD health scheme could prevent financial hardship if you get sick?

Strongly disagree: 1

Disagree: 2

Neutral: 3

Agree: 4

Strongly agree: 5

3. I trust the CKD health scheme.

Strongly disagree: 1

Disagree: 2

Neutral: 3

Agree: 4

Strongly agree: 5

4. CKD health scheme benefits are higher than the cost of HD treatment.

Strongly disagree: 1

Disagree: 2

Neutral: 3

Agree: 4

Strongly agree: 5

Part D: Preferences Regarding CKD Health Schemes

1. Currently enrolled in the CKD health scheme: 1 Yes / 2 No

If yes: a) Government b) Private c) Free d) Paid Insurance

2. Do you have previous experience with health insurance? 1 Yes / 2 No 

3. Would you prefer to pay at the time of illness instead of enrolling in CKD insurance schemes? 1 Yes / 2 No

4. Do you prefer to enroll other household members? 1 Yes / 2 No

5. Do you recommend this CKD insurance scheme to anyone? 1 Yes / 2 No

6. Are you paying an extra amount in addition to benefitted insurance? 1 Yes / 2 No

If yes, how much ________________________

Part E: Evaluation of the Scheme’s Effectiveness and the Challenges in Accessing the Benefits

1. Is this scheme’s enrolment procedure convenient for you? 1 Yes / 2 No

2. Does this scheme cover all the benefits listed below? 1 Yes / 2 No

a) Drug cost

b) Medical treatment

c) Others

3. Is this CKD health scheme fair enough to take treatment? 1 Yes / 2 No

If yes, explain.

a) Participation fees: 1 Yes / 2 No

b) Health services: 1 Yes / 2 No

c) Protection of treatment: 1 Yes / 2 No

4. How would you rate your insurance that benefits you?

Strongly disagree: 1

Disagree: 2

Neutral: 3

Agree: 4

Strongly agree: 5

5. How would you rate your overall health status at present?

Strongly disagree: 1

Disagree: 2

Neutral: 3

Agree: 4

Strongly agree: 5

6. Do you suggest any improvements to the CKD health scheme?

Questions on challenges encountered in accessing the scheme

1. Do you have knowledge about the CKD health schemes rolled out by the government?

2. Do you believe that utilizing CKD health schemes could prevent financial hardship in managing your CKD?

3. Is the CKD health scheme beneficial and simple to access?

4. CKD health scheme benefits are higher than the cost of HD treatment (rating).

5. Is this scheme's enrolment process convenient for you?

6. Does this scheme cover all the benefits listed below?

a.  Drug cost

b.  Medical treatment

c.  Others

7. Is this CKD health scheme fair enough to cover treatment costs? Specifically, on the basis of:

a.  Participation fees

b.  Health services

c.   Protection of treatment

8.  How would you rate your insurance benefits? (rating)

9.  What's your overall health management ease after using the scheme? (rating)

10.  In your opinion, which is more beneficial and effective: the government health insurance scheme or private health insurance?

11.  What options do you think could improve the use of the scheme in the state?

12.  What are the challenges you face in accessing the scheme?
